# Supplementary material for: Trace amines produced by skin bacteria accelerate wound healing in mice
Source: Commun Biol. 2020 Jun 1;3:277. doi: 10.1038/s42003-020-1000-7 (PMC7264277; doi:10.1038/s42003-020-1000-7)
Supplement: Supplementary file 1 — Supplementary Information [file 42003_2020_1000_MOESM1_ESM.pdf]

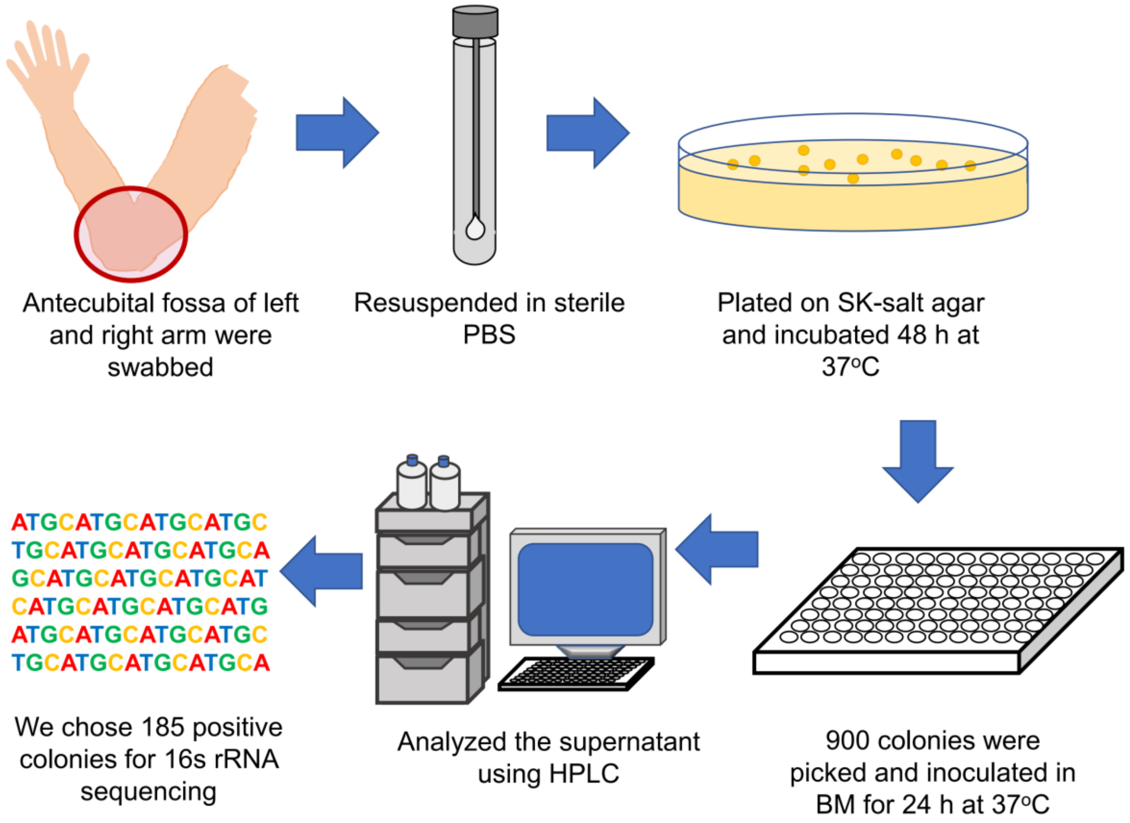

Supplementary Figure 1. Illustration of the sample collection and analyses workflow. Skin swab samples were collected from 28 volunteers and selectively grew the staphylococcal strains by spreading the swab samples on SK-salt agar (Luqman et al., 2018). The grown colonies were harvested and analyzed for TAs production by HPLC. The colonies that produce TAs were then identified using 16s rRNA sequencing.

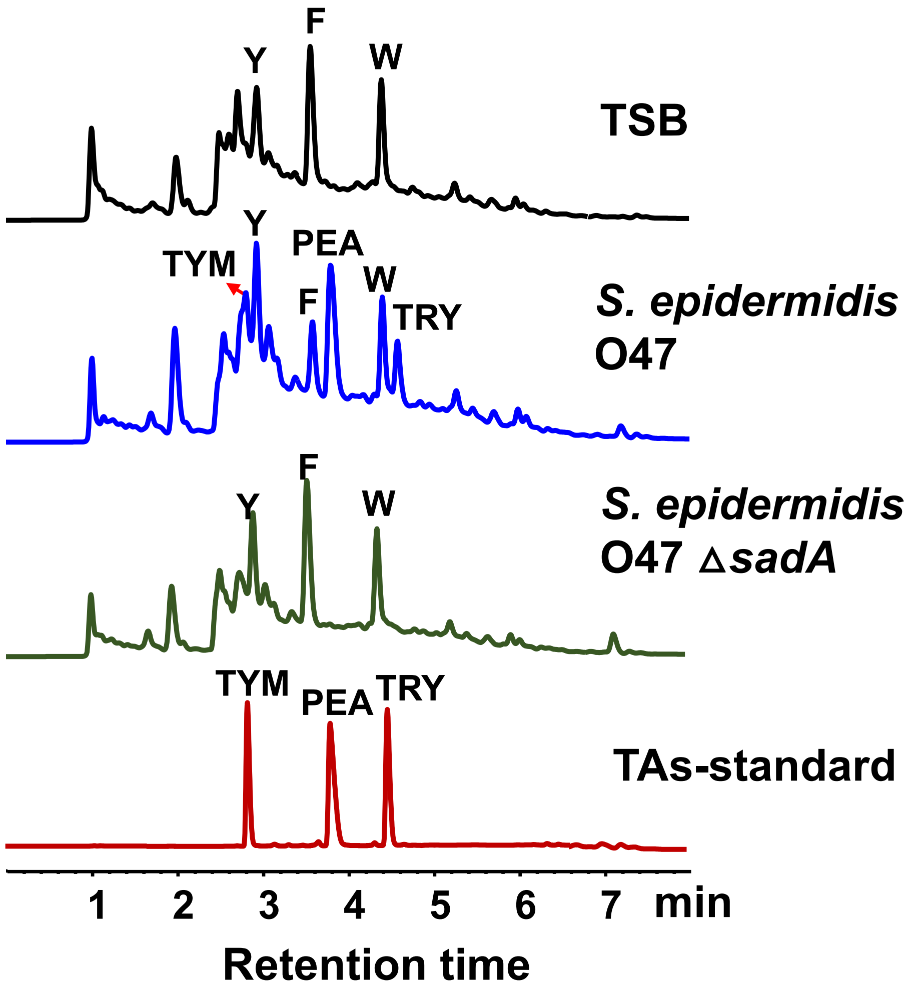

Supplementary Figure 2. HPLC-analysis of TAs production in *S. epidermidis* O47. TSB medium contains relatively high amount of AAAs (tryptophan:367, phenylalanine:613, and tyrosine:355 μg/ml) and *S. epidermidis* O47 converts approximately 20% of the AAAs into TAs (TRY:71, PEA:159, and TYM:57 μg/ml) (Luqman et al., 2018). In this study, the putative *sadA* gene in *S. epidermidis* O47 is confirmed to be responsible for the TAs production.

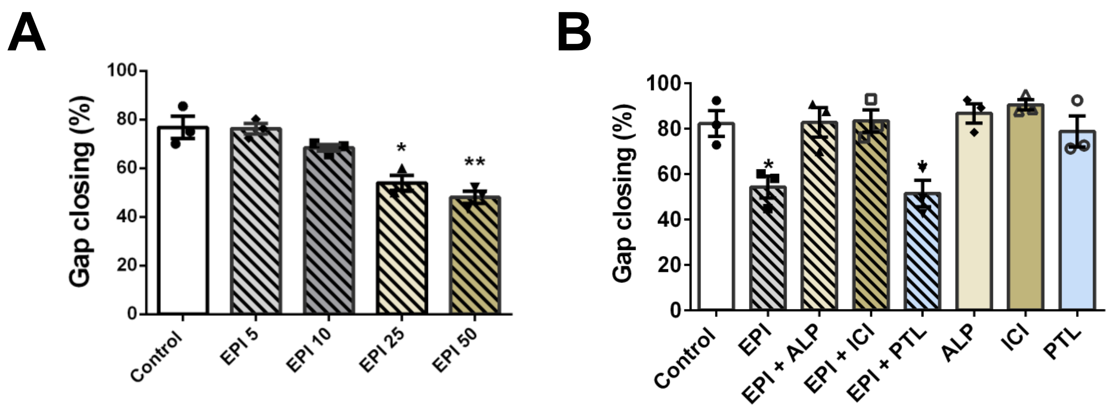

Supplementary Figure 3. Effect of compounds in *in vitro* wound healing and intracellular calcium in HaCaT cells. (A) Different concentrations of EPI were added in seeded HaCaT cells and the gap closing was measured after 24 h. **(B)** We also tested alprenolol (ALP, a neutral ß2-AR) in comparison with ICI and phentolamine (PTL, an α-AR antagonist) to examine the role of α2-AR in HaCaT cells in gap closing. For all graphs, each data point is the mean value ± SEM of 3 independent replications, *$p < 0.05$ and **$p < 0.01$, data were analyzed using paired Students t-test. Source data are provided as a Source Data file.

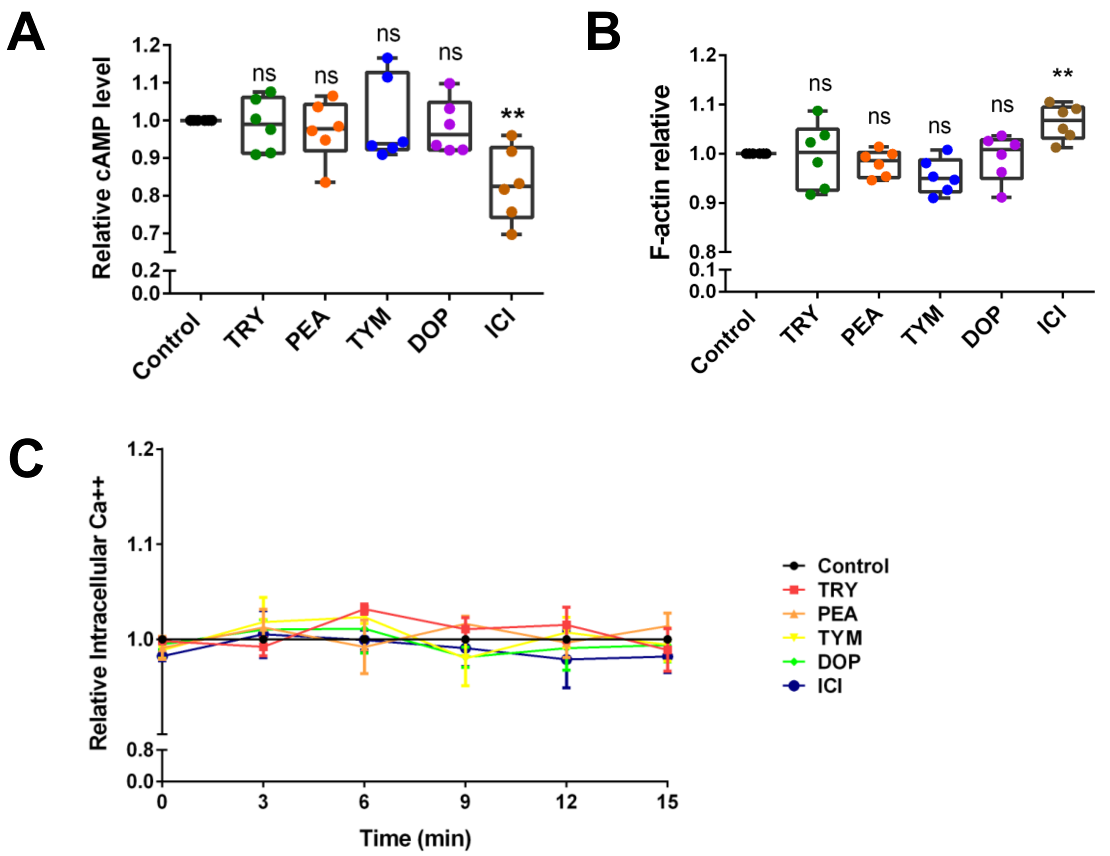

Supplementary Figure 4. Effect of TAs and DOP on HaCaT cells in the absence of EPI. We incubated HaCaT cells with TAs, DOP, and ICI (25 μg/ml) and investigated the effects on **(A)** cAMP level, **(B)** F-actin and **(C)** intracellular CA++ level. For all graphs, each data point is the mean value ± SEM from 6 independent replications for **(A & B)** and 3 independent replications for **(C)**, *p < 0.05 and **p < 0.01, data were analyzed using Students t-test. Source data are provided as a Source Data file.

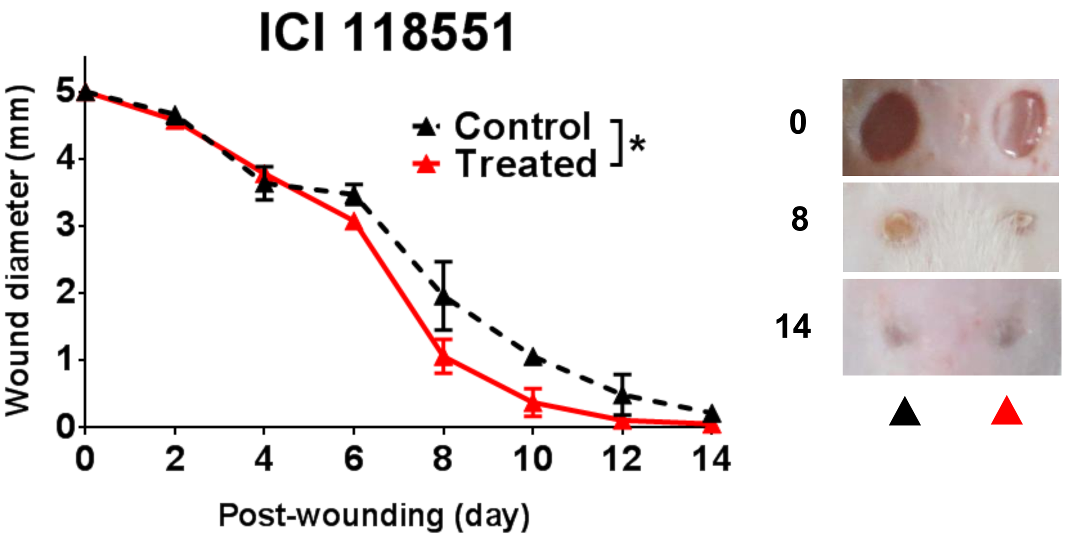

Supplementary Figure 5. Effect of ICI on wound healing in mice. Mice were shaved and wounded on their back with 2 biopsy punches for each mouse. ICI (each 25 μg/ml) with a total volume of 10 μl was applied topically and daily. The diameter of the wounds was measured every 2 days. The wounds treated with ICI showed a significantly faster wound closing than the untreated wounds (control). For all graphs, each data point is the mean value ± SEM from 3 independent replications, *p < 0.05 and **p < 0.01, data were analyzed using paired Students t-test. Source data are provided as a Source Data file.

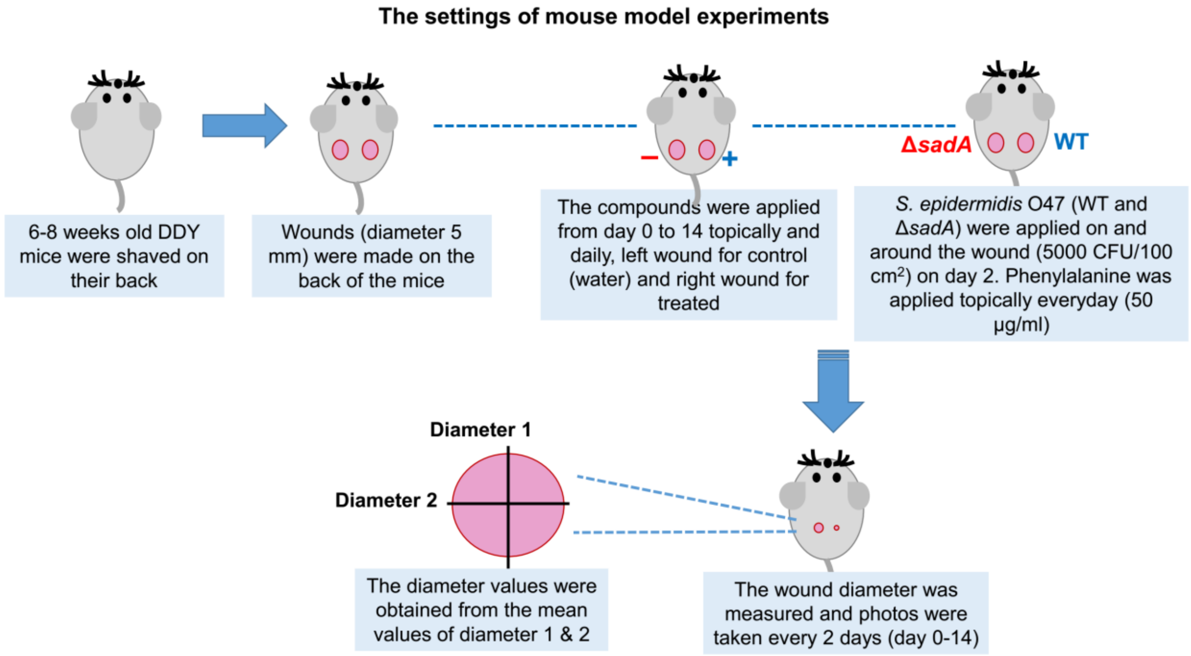

Supplementary Figure 6. The setting of mouse model experiments. 6-8 weeks old DDY mice were used for the experiments. Two wounds were made on the back of each mouse and treated with the compounds daily (10 µl at 25 µg/ml compound) with left as a control (water) and right as treated. For the treatment with bacteria, *S. epidermidis* O47 WT was applied on the right wound and Δ*sadA* on the left wound on day 2 post wounding at cell density 5000 CFU/100 cm^2 on and around the wound. Phenylalanine was applied daily (10 µl at 25 µg/ml compound). The measurements of wound diameter were performed every 2 days from two diameter values to obtain the mean values.

Supplementary Table 1. AAA and TA quantification of the skin swab samples.

Volunteers	Aromatic amino acid			Trace amine		
	Y	F	W	TYM	PEA	TRY
1	5,51	2,99	3,31	2,16	nd	0,55
2	2,79	2,3	3,17	1,92	nd	0,62
3	3,3	2,32	3,13	3,53	0,68	3,47
4	2,48	2,14	nd	3,38	nd	nd
5	nd	nd	nd	5,43	nd	nd
6	nd	nd	3,67	nd	nd	1,48
7	nd	nd	3,18	2,54	0,21	0,99
8	3,72	1,93	3,18	2,03	nd	0,56
9	nd	nd	nd	2,56	nd	nd
10	nd	nd	nd	2,54	nd	nd
11	5,58	nd	nd	nd	nd	nd
12	nd	2,05	3,09	1,88	nd	1,55
13	nd	nd	nd	2,54	nd	nd
14	nd	nd	nd	3,04	nd	1,6
15	2,68	2,08	nd	2,42	nd	nd
16	nd	nd	nd	3,06	nd	nd
17	3,34	1,93	nd	5,95	nd	nd
18	nd	nd	nd	3,03	nd	nd
19	nd	nd	3,09	2,15	nd	nd
20	nd	2,26	nd	2,37	nd	0,48
21	nd	2,31	nd	2,28	nd	nd
22	nd	2,47	nd	2,52	nd	nd
23	4,35	2,15	3,06	2,72	nd	0,56
24	2,96	2,16	3,08	1,73	nd	nd
25	4,46	2,14	3,2	1,89	nd	2,49
26	2,07	2,27	3,2	2,51	0,15	2,18
27	nd	nd	3,24	2,15	nd	nd
28	3,86	2,48	3,27	3,04	1,05	2,94

The values shown are in µg/100 cm^2. Nd:not detectable. Y:tyrosine, F: Phenylalanine, W: tryptophan, TYM: tyramine, PEA: phenethylamine, TRY: tryptamine.

Supplementary Table 2. Primers

Primer	Sequence (5'-3')
**27F**	AGAGTTTGATCCTGGCTCAG
**1392R**	GGTTACCTTGTTACGACTT
**upF**	CGCGCAGATCTGTCGACGATCCTTTTATTTTAAGAATATCTATTTATGTC
**upR**	ATAATTTCATTTCCATTTCCATACTCTCTC-
**downF**	GGAAATGGAAATGAAATTATCGAGCGCATTAAATAC
**downR**	TGCAGGCATGCAAGCTTGATAGGAAAAAAATTCACCTTAATT
