# Supplementary material for: Trace amines produced by skin bacteria accelerate wound healing in mice
Source: Commun Biol. 2020 Jun 1;3:277. doi: 10.1038/s42003-020-1000-7 (PMC7264277; doi:10.1038/s42003-020-1000-7)
Supplement: Supplementary file 2 — Description of Additional Supplementary Files [file 42003_2020_1000_MOESM2_ESM.pdf]

Description of Additional Supplementary Files

File Name: Supplementary Data 1
Description: Source data of all the graphs.

File Name: Supplementary Data 2
Description: 16s rDNA sequence data.
