# Supplementary material for: Trace amines produced by skin bacteria accelerate wound healing in mice
Source: Commun Biol. 2020 Jun 1;3:277. doi: 10.1038/s42003-020-1000-7 (PMC7264277; doi:10.1038/s42003-020-1000-7)
Supplement: Supplementary file 6 — Reporting Summary [file 42003_2020_1000_MOESM6_ESM.pdf]

nature research

Corresponding author(s): Friedrich Götz
Last updated by author(s): May 8, 2020

Reporting Summary

Nature Research wishes to improve the reproducibility of the work that we publish. This form provides structure for consistency and transparency in reporting. For further information on Nature Research policies, see Authors & Referees and the Editorial Policy Checklist.

Statistics

For all statistical analyses, confirm that the following items are present in the figure legend, table legend, main text, or Methods section.

n/a	Confirmed	
☐	☒	The exact sample size (n) for each experimental group/condition, given as a discrete number and unit of measurement
☐	☒	A statement on whether measurements were taken from distinct samples or whether the same sample was measured repeatedly
☐	☒	The statistical test(s) used AND whether they are one- or two-sided<br>*Only common tests should be described solely by name; describe more complex techniques in the Methods section.*
☒	☐	A description of all covariates tested
☐	☒	A description of any assumptions or corrections, such as tests of normality and adjustment for multiple comparisons
☐	☒	A full description of the statistical parameters including central tendency (e.g. means) or other basic estimates (e.g. regression coefficient) AND variation (e.g. standard deviation) or associated estimates of uncertainty (e.g. confidence intervals)
☐	☒	For null hypothesis testing, the test statistic (e.g. F, t, r) with confidence intervals, effect sizes, degrees of freedom and P value noted<br>*Give P values as exact values whenever suitable.*
☒	☐	For Bayesian analysis, information on the choice of priors and Markov chain Monte Carlo settings
☒	☐	For hierarchical and complex designs, identification of the appropriate level for tests and full reporting of outcomes
☒	☐	Estimates of effect sizes (e.g. Cohen's d, Pearson's r), indicating how they were calculated

Our web collection on statistics for biologists contains articles on many of the points above.

Software and code

Policy information about availability of computer code

Data collection	Homologs of SadA were collected from the tblastn of NCBI's Microbial BLAST.
Data analysis	Statistical data were analysed using GraphPad Prism 5.0.

For manuscripts utilizing custom algorithms or software that are central to the research but not yet described in published literature, software must be made available to editors/reviewers. We strongly encourage code deposition in a community repository (e.g. GitHub). See the Nature Research guidelines for submitting code & software for further information.

Data

Policy information about availability of data

All manuscripts must include a data availability statement. This statement should provide the following information, where applicable:

- Accession codes, unique identifiers, or web links for publicly available datasets
- A list of figures that have associated raw data
- A description of any restrictions on data availability

The 16s sequences data are uploaded to NCBI with accession codes MT445230-MT445414

Field-specific reporting

Please select the one below that is the best fit for your research. If you are not sure, read the appropriate sections before making your selection.

☒ Life sciences ☐ Behavioural & social sciences ☐ Ecological, evolutionary & environmental sciences

For a reference copy of the document with all sections, see nature.com/documents/nr-reporting-summary-flat.pdf

Life sciences study design

All studies must disclose on these points even when the disclosure is negative.

Sample size	We collected the human skin swab samples from 28 people.<br>We used 3 mice as a minimum number for mice experiments.
Data exclusions	No data were excluded for analysis.
Replication	Data measurements were done independently for each replicate.
Randomization	Participants for skin swab collection were chosen randomly.
Blinding	Wound diameter on mice experiments measurements were blinded.

Reporting for specific materials, systems and methods

We require information from authors about some types of materials, experimental systems and methods used in many studies. Here, indicate whether each material, system or method listed is relevant to your study. If you are not sure if a list item applies to your research, read the appropriate section before selecting a response.

Materials & experimental systems

n/a	Involved in the study
☒	☐ Antibodies
☐	☒ Eukaryotic cell lines
☒	☐ Palaeontology
☐	☒ Animals and other organisms
☐	☒ Human research participants
☒	☐ Clinical data

Methods

n/a	Involved in the study
☒	☐ ChIP-seq
☒	☐ Flow cytometry
☒	☐ MRI-based neuroimaging

Eukaryotic cell lines

Policy information about cell lines

Cell line source(s)	Department of Dermatology at the University of Tübingen
Authentication	Professor Birgit Schittek
Mycoplasma contamination	We confirmed that the cell line we used is not contaminated by Mycoplasma.
Commonly misidentified lines<br>(See ICLAC register)	N/A

Animals and other organisms

Policy information about studies involving animals; ARRIVE guidelines recommended for reporting animal research

Laboratory animals	We used DDY mice for the in vivo wound experiments.
Wild animals	The study did not involve wild animals.
Field-collected samples	The study did not involve samples collected from the field.
Ethics oversight	Ethic Commission of Faculty of Veterinary Medicine, Universitas Airlangga, Surabaya, Indonesia (Approval no. 2.KE.172.09.2019).

Note that full information on the approval of the study protocol must also be provided in the manuscript.

Human research participants

Policy information about studies involving human research participants

Population characteristics	We collected skin swabb samples from random people with no criteria.
Recruitment	Colleagues from our institute and people from outside to make sure that the participants are representative.

Ethics oversight	Ethic Commission of the University of Tübingen (Approval no. 320/2017BO2)

Note that full information on the approval of the study protocol must also be provided in the manuscript.
